# Genetic prediction of blood metabolites mediating the relationship between gut microbiota and Alzheimer’s disease: a Mendelian randomization study

**DOI:** 10.3389/fmicb.2024.1414977

**Published:** 2024-08-19

**Authors:** Guanglei Chen, Yaxian Jin, Cancan Chu, Yuhao Zheng, Yunzhi Chen, Xing Zhu

**Affiliations:** ^1^Guizhou University of Traditional Chinese Medicine, Guiyang, Guizhou, China; ^2^The First Affiliated Hospital of Guizhou University of Traditional Chinese Medicine, Guiyang, Guizhou, China

**Keywords:** gut microbiota, blood metabolites, Mendelian randomization, two-step Mendelian randomization, univariable Mendelian randomization, multivariable Mendelian randomization

## Abstract

**Background:**

Observational studies have suggested an association between gut microbiota and Alzheimer’s disease (AD); however, the causal relationship remains unclear, and the role of blood metabolites in this association remains elusive.

**Purpose:**

To elucidate the causal relationship between gut microbiota and AD and to investigate whether blood metabolites serve as potential mediators.

**Materials and methods:**

Univariable Mendelian randomization (UVMR) analysis was employed to assess the causal relationship between gut microbiota and AD, while multivariable MR (MVMR) was utilized to mitigate confounding factors. Subsequently, a two-step mediation MR approach was employed to explore the role of blood metabolites as potential mediators. We primarily utilized the inverse variance-weighted method to evaluate the causal relationship between exposure and outcome, and sensitivity analyses including Contamination mixture, Maximum-likelihood, Debiased inverse-variance weighted, MR-Egger, Bayesian Weighted Mendelian randomization, and MR pleiotropy residual sum and outlier were conducted to address pleiotropy.

**Results:**

After adjustment for reverse causality and MVMR correction, class Actinobacteria (OR: 1.03, 95% CI: 1.01–1.06, *p* = 0.006), family Lactobacillaceae (OR: 1.03, 95% CI: 1.00–1.05, *p* = 0.017), genus *Lachnoclostridium* (OR: 1.03, 95% CI: 1.00–1.06, *p* = 0.019), genus *Ruminiclostridium9* (OR: 0.97, 95% CI: 0.94–1.00, *p* = 0.027) and genus *Ruminiclostridium6* (OR: 1.03, 95% CI: 1.01–1.05, *p* = 0.009) exhibited causal effects on AD. Moreover, 1-ribosyl-imidazoleacetate levels (−6.62%), Metabolonic lactone sulfate levels (2.90%), and Nonadecanoate (19:0) levels (−12.17%) mediated the total genetic predictive effects of class Actinobacteria on AD risk. Similarly, 2-stearoyl-GPE (18:0) levels (−9.87%), Octadecanedioylcarnitine (C18-DC) levels (4.44%), 1-(1-enyl-stearoyl)-2-oleoyl-GPE (p-18:0/18:1) levels (38.66%), and X-23639 levels (13.28%) respectively mediated the total genetic predictive effects of family Lactobacillaceae on AD risk. Furthermore, Hexadecanedioate (C16-DC) levels (5.45%) mediated the total genetic predictive effects of genus *Ruminiclostridium 6* on AD risk; Indole-3-carboxylate levels (13.91%), X-13431 levels (7.08%), Alpha-ketoglutarate to succinate ratio (−13.91%), 3-phosphoglycerate to glycerate ratio (15.27%), and Succinate to proline ratio (−14.64%) respectively mediated the total genetic predictive effects of genus *Ruminiclostridium 9* on AD risk.

**Conclusion:**

Our mediation MR analysis provides genetic evidence suggesting the potential mediating role of blood metabolites in the causal relationship between gut microbiota and AD. Further large-scale randomized controlled trials are warranted to validate the role of blood metabolites in the specific mechanisms by which gut microbiota influence AD.

## 1 Introduction

Alzheimer’s disease (AD) is a neurodegenerative disorder characterized by cognitive impairment, memory decline, personality changes, and psychiatric symptoms. The pathology of AD typically involves the deposition of extracellular β-amyloid protein (Aβ), forming neuroinflammatory plaques, and the intracellular accumulation of hyperphosphorylated tau protein, leading to the formation of neurofibrillary tangles. These processes are accompanied by oxidative stress, inflammatory responses, ultimately resulting in neurotoxicity and neuroinflammation, leading to neuronal damage and severe cognitive impairment (Long and Holtzman, 2019). According to the World Alzheimer Report 2022, AD accounts for approximately 60–80% of all dementia cases (Gauthier et al., 2022), with nearly 55 million AD patients worldwide in 2019, a figure projected to rise to 139 million by 2050. However, the precise pathological mechanisms underlying the onset and progression of AD remain incompletely understood, and research into therapeutic modalities remains incomplete. Therefore, the exploration of alternative effective treatment approaches and drug targets holds significant importance in the prevention and treatment of AD.

The gut microbiota plays a pivotal role in human health. Mounting evidence suggests that it influences brain function and behavior via the microbiota-gut-brain axis, thereby contributing to the onset and progression of neurological disorders such as depression, Parkinson’s disease, and AD (Rogers et al., 2016). Intervention mechanisms may involve the modulation of brain inflammation pathways through inflammasome signaling (Wong et al., 2016) or the regulation of gene expression in the brain (Distrutti et al., 2014), thereby influencing neurodevelopment (Rogers et al., 2016). In addition to alterations in the composition and abundance of gut microbiota impacting AD, the gut microbiota can also influence AD by transferring metabolites to the brain via bidirectional communication along the gut-brain axis and regulating brain gene expression (Arentsen et al., 2017; Zou et al., 2024). However, the specific correlation between AD onset and the gut microbiota remains incompletely elucidated, and the mechanisms underlying the role of metabolites in the interaction between AD and gut microbiota remain unknown. Therefore, there is an urgent need for a comprehensive and in-depth analysis of the effects of metabolite-mediated gut microbiota on the genetic predisposition to and risk of AD.

Mendelian Randomization (MR) is a statistical analysis method grounded in Mendelian genetic principles and instrumental variables (IVs). It effectively reduces confounding factors by utilizing genetic data as intermediaries to explore causal relationships between exposure risk factors and outcomes (Davey Smith and Ebrahim, 2003). Our objective here is to employ univariate Mendelian randomization (UVMR), multivariate Mendelian randomization (MVMR), and MR-based mediation analysis across two samples to dissect the causal impact of gut microbiota on AD and ascertain the mediating role of metabolites.

In selecting single nucleotide polymorphisms (SNPs) as IVs, several conditions must be met: (a) the relevance assumption, wherein each IV must be strongly associated with the exposure factor; (b) the independence assumption, meaning each IV must not be associated with other potential confounders; (c) the exclusion restriction, indicating each IV can only influence the outcome through the exposure factor.

## 2 Materials and methods

### 2.1 Ethical considerations

For this study, we adhered to a comprehensive “Strengthening the Reporting of Observational studies in Epidemiology-Mendelian Randomization (STROBE-MR) statement” (Skrivankova et al., 2021).

### 2.2 Study design

**Figure 1:**
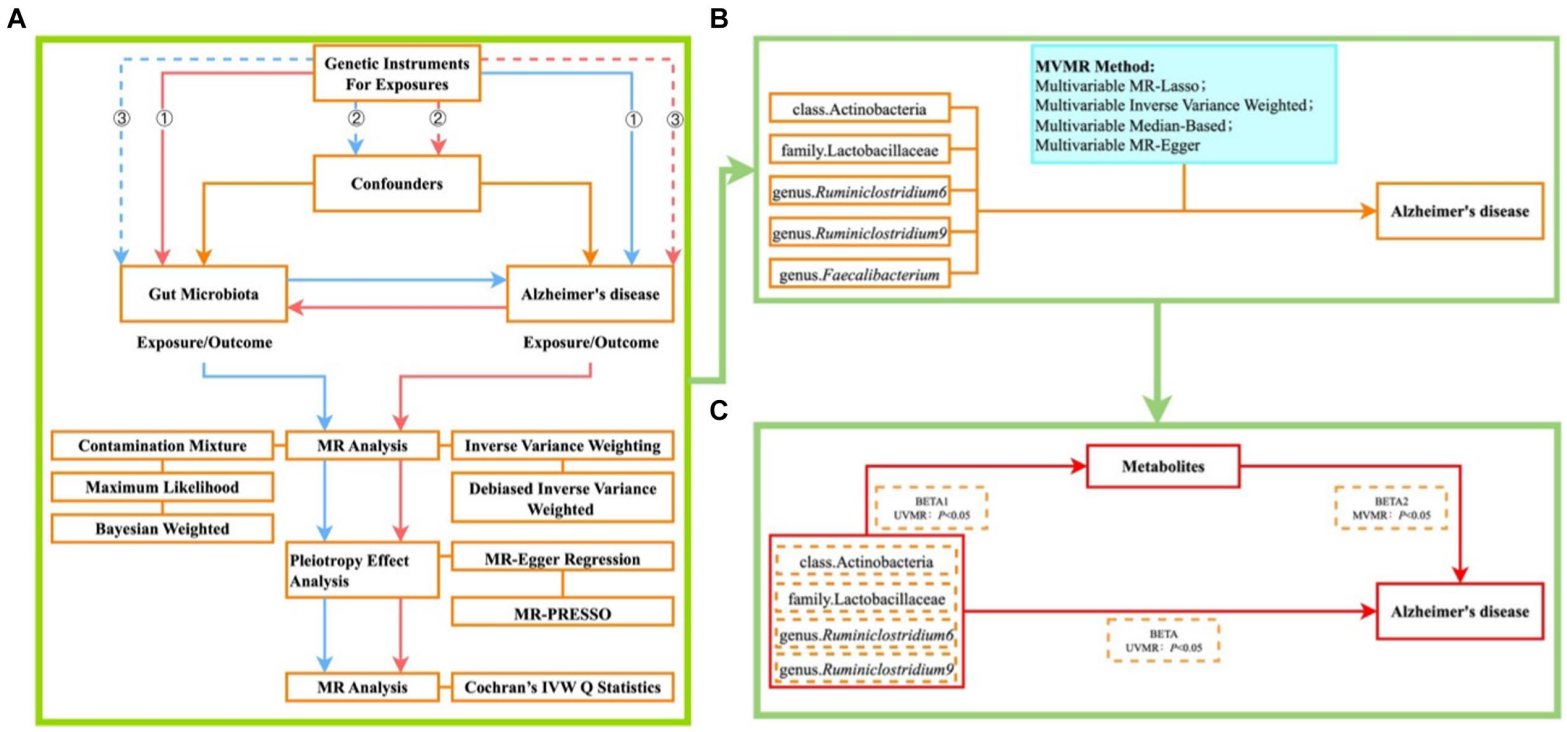
Illustrates the MR study design flowchart **(A)** ① each IV significantly associated with exposure; ② each IV unaffected by confounding factors to reduce bias caused by LD; ③ each IV only influences the outcome through exposure; blue lines represent forward MR analysis between gut microbiota as exposure and AD as the outcome; red lines represent reverse MR analysis between AD as exposure and gut microbiota as the outcome. **(B)** Plot of MVMR analysis of positive bacteria versus AD; **(C)** Plot of metabolite-mediated mediator analysis of gut microbiota effects on AD.

The design of our MR study is illustrated in Figure 1. Initially, we investigated the forward UVMR analysis between gut microbiota as exposure and Alzheimer’s disease (AD) as the outcome, and through reverse UVMR analysis between AD as exposure and gut microbiota as the outcome, we excluded gut microbiota with reverse causality (Figure 1A). The five positively associated gut microbiota identified were then adjusted using MVMR to obtain gut microbiota with a causal effect on AD (Figure 1B). Finally, employing a two-step method (Yuan et al., 2022), we explored the cumulative genetic predisposition impact of metabolites mediating the relationship between gut microbiota and AD risk. In the first step, gut microbiota and metabolites underwent conventional UVMR analysis to obtain BETA1 (*p* < 0.05). In the second step, the metabolites obtained from the first step were subjected to MVMR analysis with gut microbiota to obtain BETA2 (*p* < 0.05). At this stage, the UVMR analysis between gut microbiota and AD yielded the total effect BETA, with the mediation effect represented by BETA1*BETA2 and the direct effect as BETA-BETA1*BETA2. The proportion of the mediation effect was calculated as BETA1*BETA2/BETA (Figure 1C).

### 2.3 Data sources

#### 2.3.1 Alzheimer’s disease

The GWAS data for AD were obtained from the Psychiatric Genomics Consortium (PGC) database, derived from a three-stage Meta-Analysis led by Jansen et al. (2019). This analysis included clinically diagnosed late-onset AD cases and proxy cases in European populations, confirming 29 risk loci and 215 potential pathogenic genes, thereby elucidating the genetic factors underlying AD.

#### 2.3.2 Gut microbiota

Data on gut microbiota were sourced from the MiBioGen database, one of the most comprehensive host-genetic-microbiome association databases to date. This database comprises 24 population cohorts spanning 11 countries and multiple ethnicities, totaling 18,340 participants (MiBioGen Consortium Initiative et al., 2018; Kurilshikov et al., 2021), all of whom are of European descent (Li et al., 2023).

#### 2.3.3 Metabolites

The GWAS data for metabolites were obtained from metabolomic studies led by Chen et al. (2023). This study, based on the Canadian Longitudinal Study on Aging (CLSA) cohort, encompassed 8,299 individuals and analyzed 1,091 blood metabolites and 309 metabolite ratios. Among these metabolites, 850 were categorized as known metabolites, including lipids, amino acids, exogenous substances, nucleotides, cofactors, vitamins, carbohydrates, peptides, and energy, while the remaining 241 were classified as unknown metabolites. Given that many metabolites serve as substrates and products of enzyme-catalyzed reactions simultaneously, identifying the genetic determinants of substrate-to-product ratios can provide insights into biological processes that cannot be gleaned from studying individual metabolites alone. Consequently, a genome-wide association study was conducted on 309 metabolite ratios to identify novel associations between genetic variations and metabolite ratios.

### 2.4 Instrumental variable selection

When employing gut microbiota as the exposure factor and AD as the outcome, stringent criteria were applied to IVs to ensure the stability of study data and the accuracy of results. These criteria were as follows: (a) IVs associated with gut microbiota at a genome-wide significance threshold of *p* < 1 × 10^−5^ (Cheng et al., 2024); (b) To meet the conditions of MR analysis, linkage disequilibrium (LD) analysis based on the European Thousand Genomes Project was conducted, requiring an *R*^2^ < 0.001 and LD = 10,000 kb for IVs; (c) To mitigate the potential impact of allele bias on the causal relationship between gut microbiota and AD, the strength of genetic variation serving as IVs was assessed using the F-statistic. Variants with an F-statistic ≤10 were considered weak IVs, likely to bias the analysis results. Conversely, an F-statistic >10 indicated robust instrumental variables, hence IVs with an F-statistic less than 10 were excluded (Burgess et al., 2017). Additionally, for reverse MR analysis, IVs for AD were required to meet the following criteria: *p* < 5 × 10^−8^, *R*^2^ < 0.001, LD = 10,000 kb, and IVs with an F-statistic less than 10 were similarly excluded (see Supplementary materials S1, S2).

During the two-step mediation MR and MVMR analyses, instrumental variables for metabolites were subject to the following criteria: *p* < 1 × 10^−5^, *R*^2^ < 0.001, LD = 10,000 kb, and IVs with an F-statistic less than 10 were also excluded (see Supplementary material S3).

### 2.5 Statistical analysis

We obtained the required data from publicly available databases including PGC, MiBioGen, and Catalog GWAS. Subsequently, a bidirectional MR analysis was conducted to investigate the causal relationship between gut microbiota and AD, while simultaneously excluding gut microbiota with reverse causality concerning AD. Following this, MVMR was employed to adjust for gut microbiota, and finally, a two-step mediation MR analysis was performed to explore the cumulative genetic predisposition impact of human blood metabolites mediating the relationship between gut microbiota and AD risk. During the MR analysis, R (version 4.3.1) was primarily utilized, assisted by the “Two Sample MR” R package (version 0.5.7) (Mounier and Kutalik, 2023), “Mendelian Randomization” R package (version 0.9.0), and “BWMR” R package (version 0.1.1) (Zhao et al., 2020).

The coefficient of determination (R^2^) was employed to indicate the proportion of phenotypic variance explained by SNPs, calculated as $R^{2}=\frac{2\times {\mathrm{Beta}}^{2}\times \mathrm{EAF}\times \left(1-\mathrm{EAF}\right)}{2\times {\mathrm{Beta}}^{2}\times \mathrm{EAF}\times \left(\begin{array}{l} 1-\\ \mathrm{EAF} \end{array}\right)+{\mathrm{SE}}^{2}\times 2\times \mathrm{Sample size}\times \mathrm{EAF}\times \left(\begin{array}{l} 1-\\ \mathrm{EAF} \end{array}\right)}$. To assess the strength of instrumental variables (IVs), the F-statistic was computed using the formula $F=\frac{R^{2}\times \left(Samplesize-1-k\right)}{\left(1-R^{2}\right)\times k}$, where R^2^ represents the proportion of phenotypic variance explained by SNPs, and k denotes the number of SNPs included in the instrument (Palmer et al., 2012). A threshold of F-statistic greater than 10 is commonly considered statistically significant, indicating that the causal relationship remains unbiased (Zuber et al., 2020).

In the UVMR analysis, we initially employed the Inverse-Variance Weighted (IVW) method to validate the effectiveness of all instrumental variables (IVs) and generate a weighted overall effect based on the magnitude of *p*-values (Brion et al., 2013). To ensure the robustness of IVW results and mitigate biases introduced by ineffective IVs, various supplementary MR analyses were conducted, including Contamination mixture, Maximum-likelihood, Debiased inverse-variance weighted, MR-Egger, Bayesian Weighted Mendelian Randomization (BWMR), and MR pleiotropy residual sum and outlier (MR-PRESSO). Although Contamination mixture MR analysis does not remove outlier IVs, it operates under the assumption that effective IVs comprise the maximal subset of all IVs, thereby yielding more precise causal effects than IVW results (Burgess et al., 2020). The Maximum-likelihood MR analysis method accommodates both correlated and uncorrelated genetic variations. If fixed-effects models are inappropriate in IVW and substantial heterogeneity exists in the causal effects of different variables, Maximum-likelihood MR analysis employs a random-effects model to address the heterogeneity present (Burgess et al., 2013). In instances where weak IVs are inevitable, Debiased inverse-variance weighted method is employed for MR analysis. This approach demonstrates robustness against many weak IVs and requires no pre-selection (Ye et al., 2021). BWMR considers the uncertainty associated with weak effects induced by multiple genes and detects outliers through Bayesian Weighted, thereby addressing violations of MR assumptions caused by multi-gene effects (Zhao et al., 2020). Finally, we conducted a series of sensitivity analyses, including MR-Egger regression, MR-PRESSO analysis, and Cochran’s *Q*-test. MR-Egger regression (Zhang et al., 2024) was utilized to detect directional pleiotropy by estimating the intercept term. If the *p*-value of the intercept is greater than 0.05, it indicates the absence of significant directional pleiotropy, thereby suggesting that the SNPs used do not influence the outcome variable through alternative pathways, thus supporting the robustness of our findings. The MR-PRESSO test (Lou et al., 2023) was employed to identify and correct for horizontal pleiotropy, not only detecting pleiotropic outliers but also adjusting the results by iteratively removing these outliers. Lastly, Cochran’s *Q*-test (Zheng X. et al., 2023) was used to assess heterogeneity among the instrumental variables. A p-value less than 0.05 for the Q statistic indicates significant heterogeneity, necessitating the use of a random-effects model to adjust the results.

In MVMR, we employed the Multivariable IVW method to ascertain the effectiveness of all IVs, thereby generating a weighted overall effect based on the significance of *p*-values (Brion et al., 2013). Additionally, Multivariable median-based estimation enabled precise assessment of causal relationships. Furthermore, Multivariable MR-Egger was utilized to evaluate whether genetic variations exhibit pleiotropy, affecting outcomes differing from zero on average. This approach involves directional pleiotropy tests, causal effect tests, and causal effect estimates, providing consistent estimates of causal effects under the weaker InSIDE assumption (Burgess and Thompson, 2017). Finally, Multivariable MR-Lasso was employed to introduce intercept terms for each genetic variation to extend the IVW model. This technique repositions genetic variations through regularized regression to identify effective instrumental variables, utilizing only these variables for IVW estimation of causal effects (Rees et al., 2019).

## 3 Results

### 3.1 Causal effects of gut microbiota on AD

**Figure 2:**
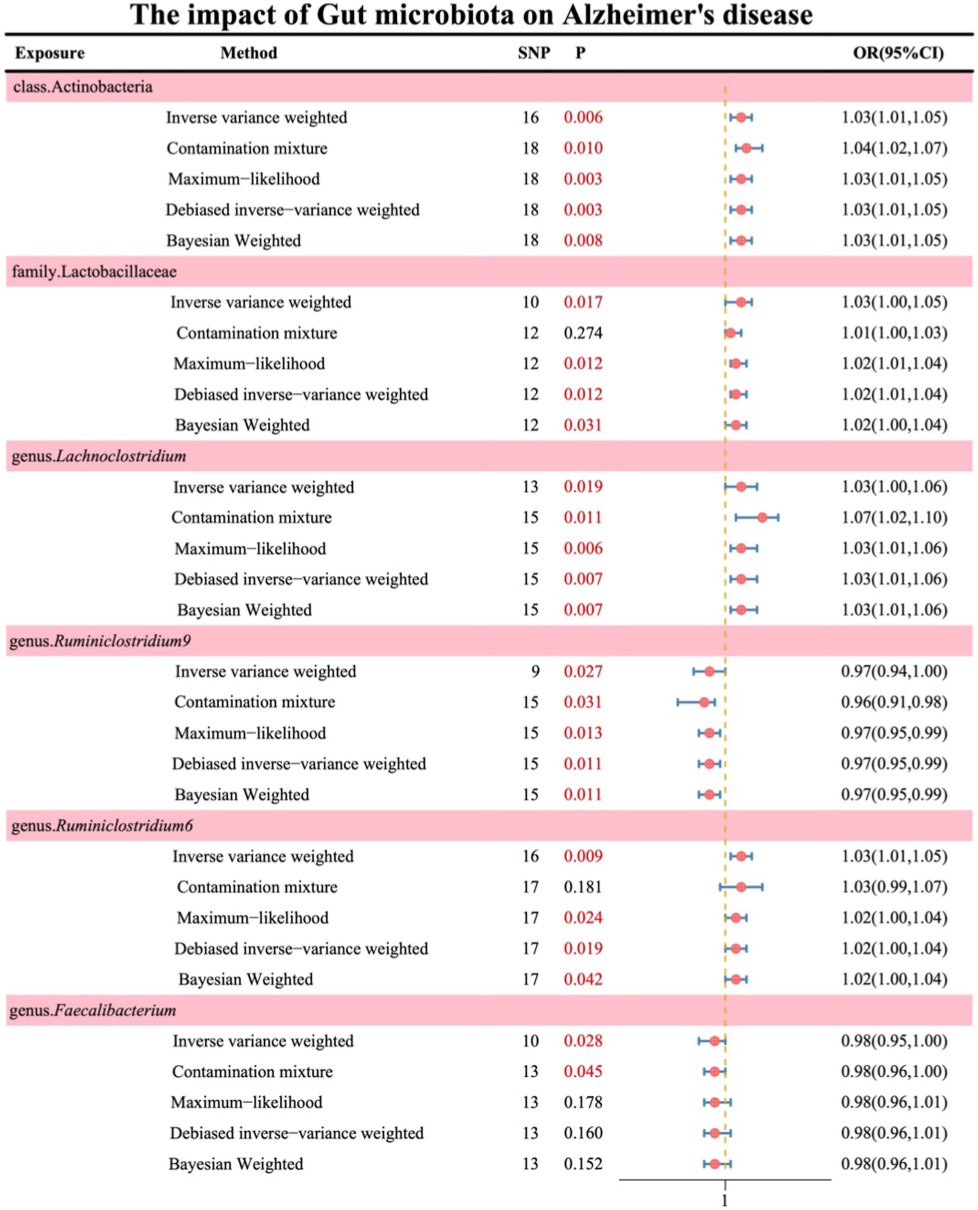
Displays the causal effects of gut microbiota on AD as analyzed through UVMR.

Following the absence of significant heterogeneity as indicated by Cochran’s Q test, initial IVW results revealed the following associations: class Actinobacteria (OR: 1.03, 95% CI: 1.01–1.06, *p* = 0.006), family Lactobacillaceae (OR: 1.03, 95% CI: 1.00–1.05, *p* = 0.017), genus *Lachnoclostridium* (OR: 1.03, 95% CI: 1.00–1.06, *p* = 0.019), genus *Ruminiclostridium9* (OR: 0.97, 95% CI: 0.94–1.00, *p* = 0.027), genus *Ruminiclostridium6* (OR: 1.03, 95% CI: 1.01–1.05, *p* = 0.009), and genus *Faecalibacterium* (OR: 0.98, 95% CI: 0.95–1.00, *p* = 0.028). The results of the remaining four methods are illustrated in Figure 2 (see Supplementary material S4).

Simultaneously, in the sensitivity analysis, we performed MR-Egger regression, Cochran’s Q test, and MR-PRESSO test (Table 1), with all results remaining stable (see Supplementary material S4 for details).

**Table 1:** Sensitivity Analysis of the causal effect of gut microbiota on AD.

Exposure	Outcome	MR egger regression			Cochran’s Q			MR-PRESSO global test P
		MR egger intercept	Intercept SE	Intercept P	Q	Q df	Q Pval	
class.Actinobacteria	AD	−0.004	0.002	0.071	18.310	15	0.247	0.235
family.Lactobacillaceae		0.005	0.003	0.152	12.909	9	0.167	0.194
genus.Lachnoclostridium		0.000	0.003	0.982	13.195	12	0.355	0.378
genus.Ruminiclostridium9		0.001	0.004	0.879	7.386	8	0.496	0.514
genus.Ruminiclostridium6		0.002	0.002	0.406	16.425	15	0.354	0.391
genus.Faecalibacterium		0.000	0.002	0.853	7.594	9	0.576	0.667

### 3.2 Causal effects of AD on gut microbiota

**Figure 3:**
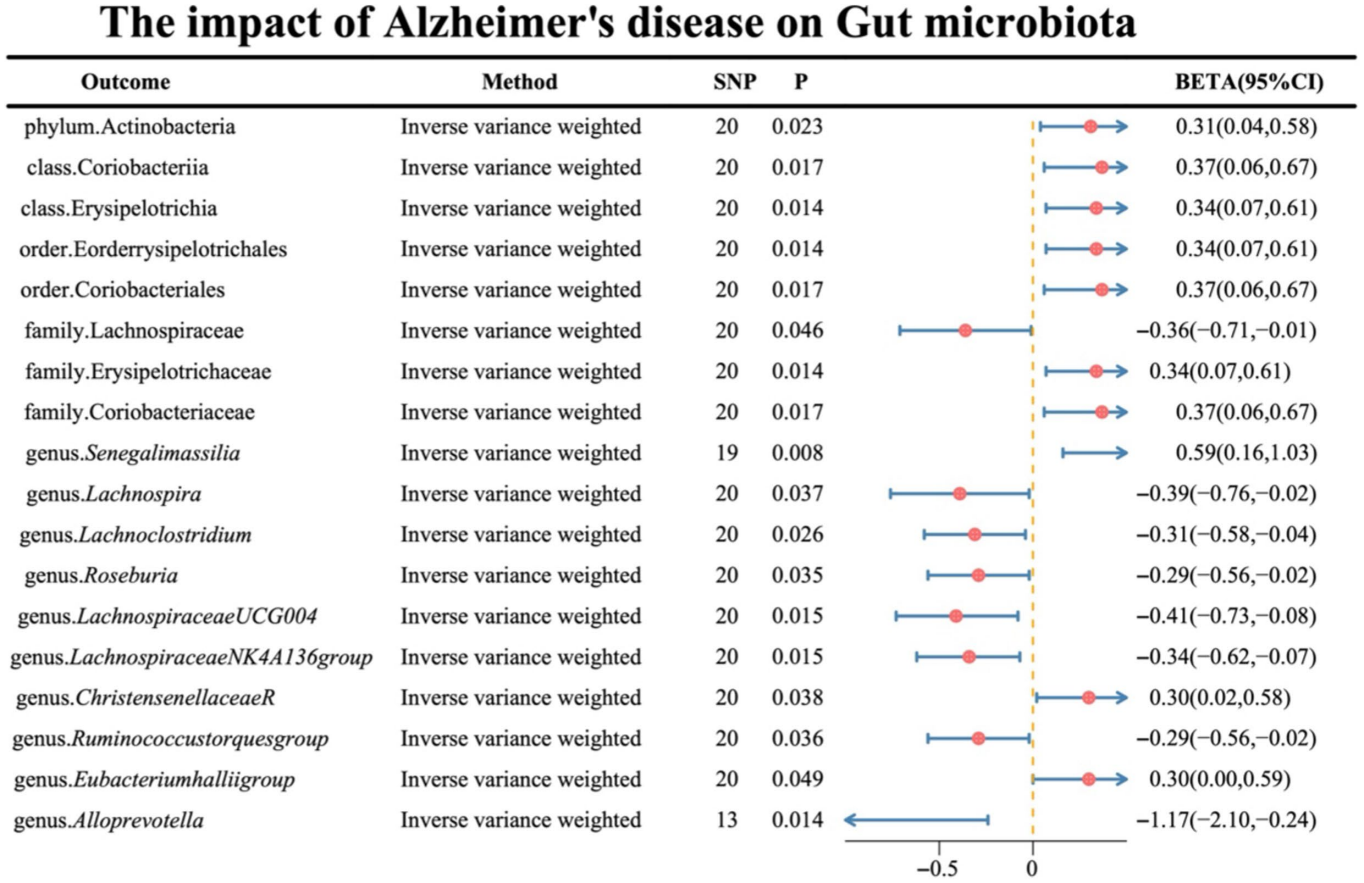
Illustrates the causal effects of AD on gut microbiota analyzed through UVMR.

Regarding the causal effects of AD on gut microbiota, the IVW results are presented in Figure 3, with the remaining four supplementary methods provided in Supplementary material S4. Notably, genus *Lachnoclostridium* exhibited a reverse causality [BETA: −0.31, 95% CI: (−0.58, −0.04), *p* = 0.026] (Supplementary material S5).

Simultaneously, in the sensitivity analysis, we performed MR-Egger regression, Cochran’s *Q*-test, and MR-PRESSO test (Table 2), with nearly all results remaining stable, except for genus *Lachnospira* and family *Lachnospiraceae*, which exhibited heterogeneity and horizontal pleiotropy. After the removal of outliers, no horizontal pleiotropy was observed (see Supplementary material S5 for details).

**Table 2:** Sensitivity Analysis of the causal effect of gut microbiota on AD.

Outcome	Exposure	MR egger regression			Cochran’s Q			MR-PRESSO Global test P	MR-PRESSO results distortion test P
		MR egger intercept	Intercept SE	Intercept P	Q	Q df	Q Pval		
phylum.Actinobacteria	AD	−0.005	0.009	0.570	18.781	19	0.471	0.506	
order.Erysipelotrichales		−0.004	0.008	0.680	13.497	19	0.812	0.794	
order.Coriobacteriales		−0.002	0.010	0.848	23.628	19	0.211	0.233	
genus.Senegalimassilia		−0.001	0.014	0.967	16.037	18	0.590	0.609	
genus.Roseburia		0.011	0.009	0.201	16.472	19	0.626	0.639	
genus.LachnospiraceaeUCG004		0.001	0.011	0.958	22.133	19	0.278	0.293	
genus.LachnospiraceaeNK4A136group		−0.002	0.009	0.825	15.105	19	0.716	0.716	
genus.Lachnospira		0.018	0.011	0.116	32.548	19	0.027	0.016	0.731
genus.Lachnoclostridium		0.001	0.009	0.879	17.045	19	0.587	0.578	
genus.ChristensenellaceaeR.7group		−0.013	0.009	0.161	11.616	19	0.901	0.863	
genus.Alloprevotella		0.028	0.047	0.569	10.830	12	0.544	0.554	
genus.Ruminococcustorquesgroup		−0.006	0.009	0.524	16.903	19	0.596	0.632	
genus.Eubacteriumhalliigroup		−0.005	0.009	0.609	20.948	19	0.340	0.312	
family.Lachnospiraceae		0.005	0.011	0.652	32.933	19	0.024	0.036	0.657
family.Erysipelotrichaceae		−0.004	0.008	0.680	13.497	19	0.812	0.814	
family.Coriobacteriaceae		−0.002	0.010	0.848	23.628	19	0.211	0.211	
class.Erysipelotrichia		−0.004	0.008	0.680	13.497	19	0.812	0.788	
class.Coriobacteriia		−0.002	0.010	0.848	23.628	19	0.211	0.229	

### 3.3 Causal effects of gut microbiota on AD (adjusted by MVMR)

After conducting both forward (where gut microbiota serves as exposure and AD as outcome) and reverse (where AD serves as exposure and gut microbiota as outcome) UVMR analyses and excluding reverse causality, gut microbiota with causal effects on AD were identified as follows: class Actinobacteria, family Lactobacillaceae, genus *Ruminiclostridium9*, genus *Ruminiclostridium6*, and genus *Faecalibacterium*. Subsequently, MVMR was employed to adjust these five types of gut microbiota. The results indicated that class Actinobacteria (OR: 1.02, 95% CI: 1.00–1.04, *p* = 0.013), family Lactobacillaceae (OR: 1.02, 95% CI: 1.01–1.04, *p* = 0.005), genus *Ruminiclostridium9* (OR: 0.97, 95% CI: 0.95–0.99, *p* = 0.007), genus *Ruminiclostridium6* (OR: 1.02, 95% CI: 1.00–1.04, *p* = 0.014), and genus *Faecalibacterium* (OR: 1.00, 95% CI: 0.98–1.02, *p* = 0.964).

**Figure 4:**
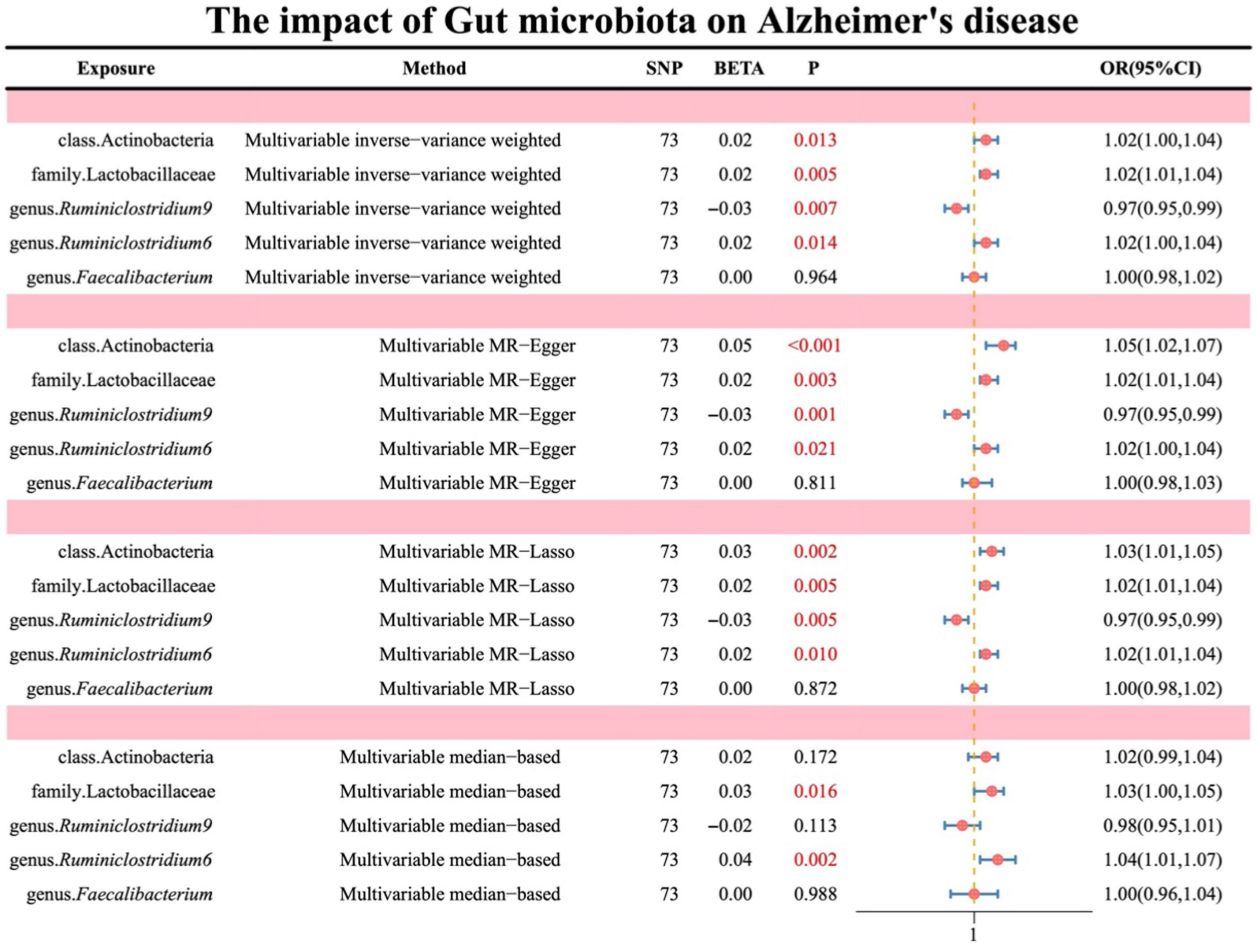
Illustrates the causal effects of gut microbiota on AD analyzed through MVMR.

Additionally, Multivariable MR-Lasso, Multivariable median-based, and Multivariable MR-Egger were utilized as supplementary methods to further elucidate the findings, all of which demonstrated consistent stability (see Figure 4; Supplementary material S6).

### 3.4 Causal effects of gut microbiota on serum metabolites

**Figure 5:**
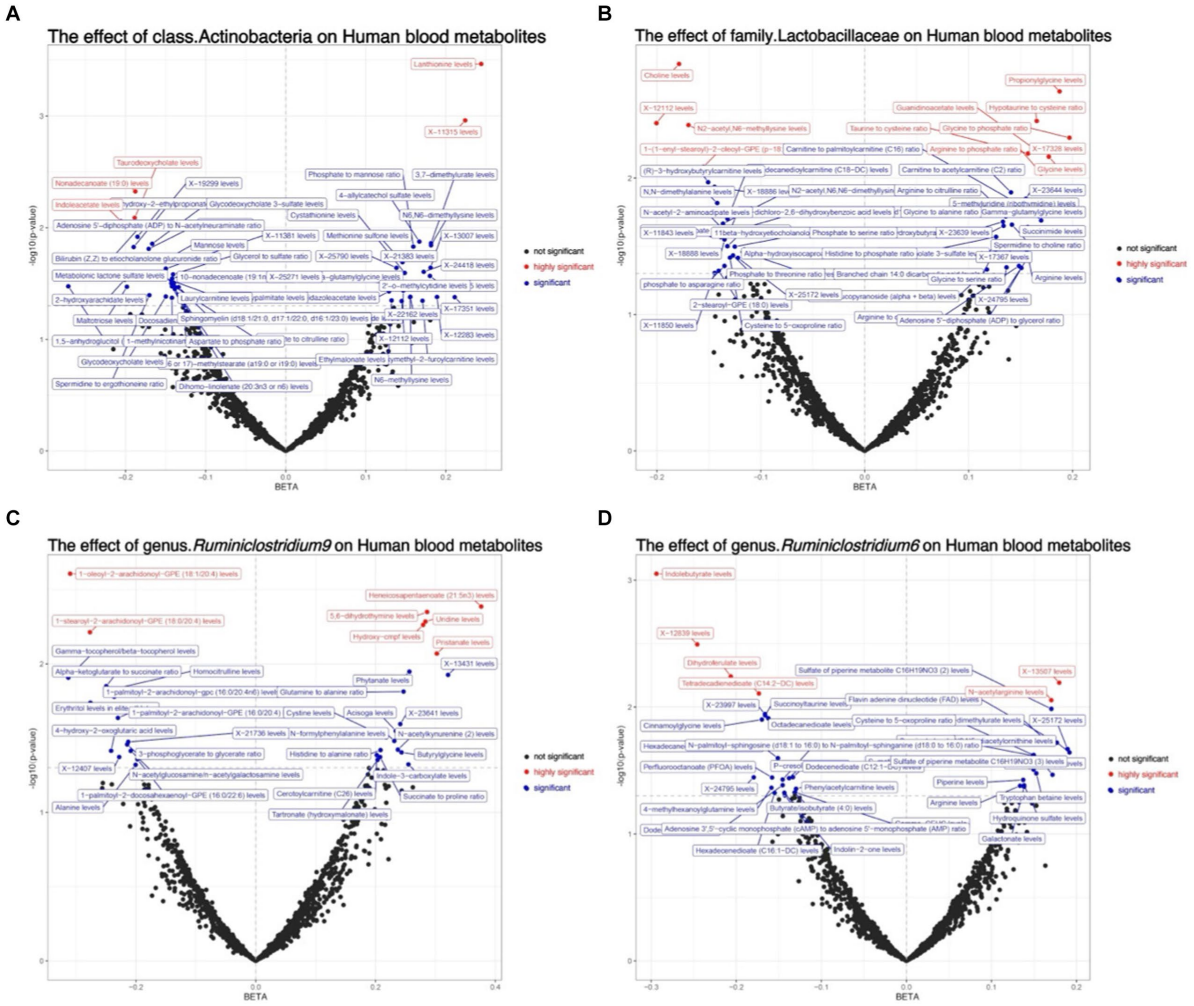
Depicts the volcano plot analysis of the causal effects of gut microbiota on metabolites, where black dots represent insignificance, red dots signify extreme significance (0 < *p* < 0.01), and blue dots indicate significance (0.01 < *p* < 0.05). **(A)** The effect of class.Actinobacteria on Human blood metabolites; **(B)** The effect of family.Lactobacillaceae on Human blood metabolites; **(C)** The effect of genus.*Ruminiclostridium9* on Human blood metabolites; **(D)** The effect of genus.*Ruminiclostridium6* on Human blood metabolites.

The primary approach employed for this investigation was IVW, with results depicted in a volcano plot (see Figure 5; Supplementary material S7).

### 3.5 Causal effects of metabolites on AD

**Figure 6:**
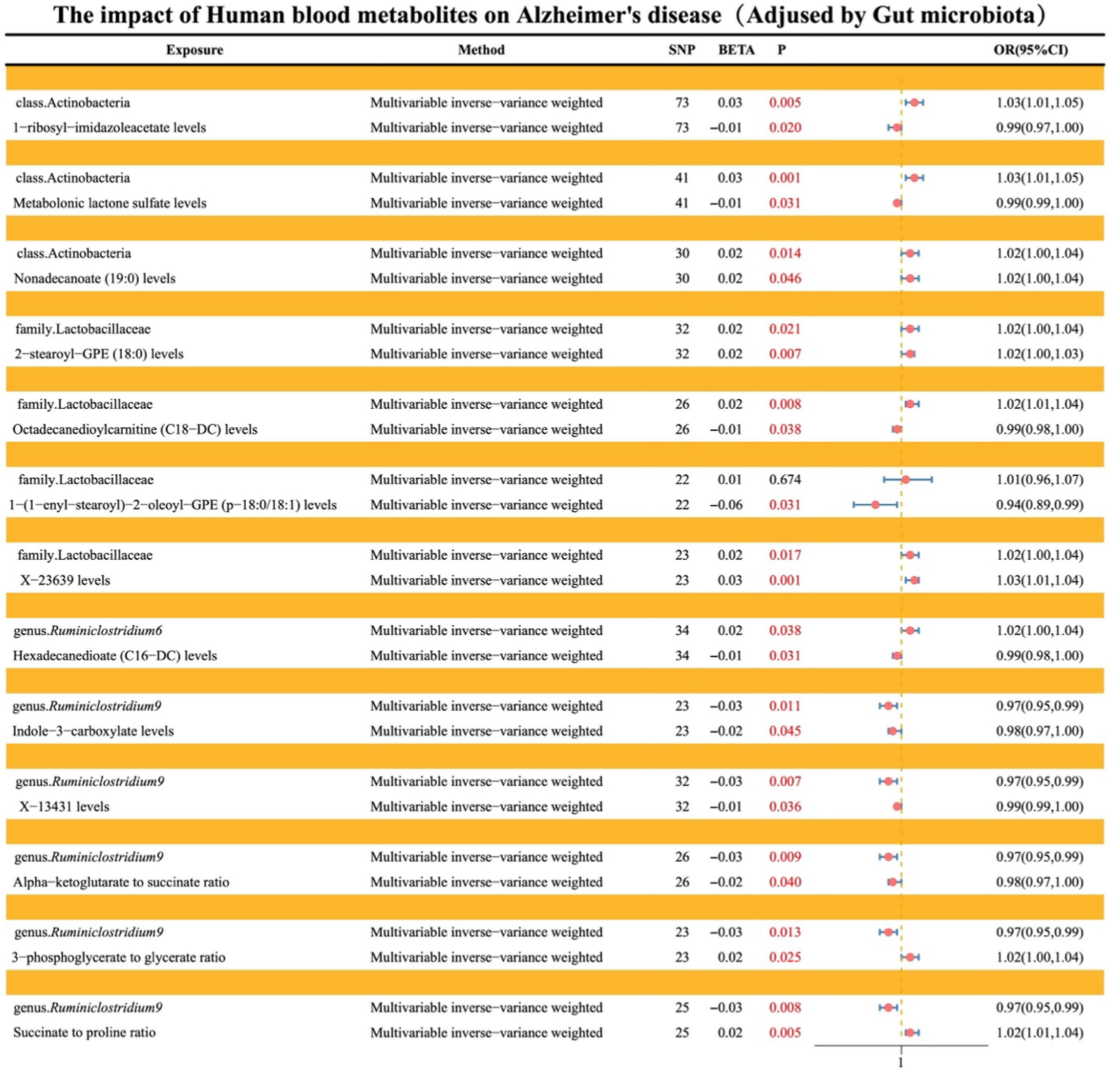
Illustrates the forest plot analysis of the causal effects of metabolites on AD.

Levels of nonadecanoate (19:0), 2-stearoyl-GPE (18:0), X-23639, 3-phosphoglycerate to glycerate ratio, and succinate to proline ratio exhibit a positive correlation with the risk of AD. Conversely, levels of 1-ribosyl-imidazoleacetate, metabolonic lactone sulfate, octadecanedioylcarnitine (C18-DC), 1-(1-enyl-stearoyl)-2-oleoyl-GPE (p-18:0/18:1), hexadecanedioate (C16-DC), indole-3-carboxylate, X-13431, and alpha-ketoglutarate to succinate ratio demonstrate a negative correlation with the risk of AD (see Figure 6; Supplementary material S8).

### 3.6 Mediation of metabolites in the genetic prediction of gut microbiota and AD

**Figure 7:**
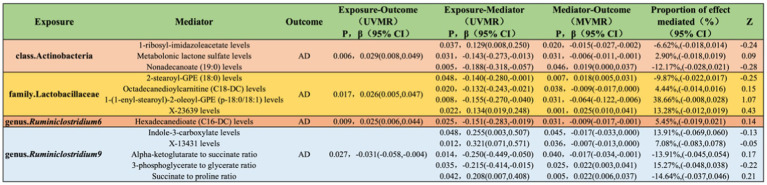
Illustrates the causal effects of gut microbiota mediated by blood metabolites in the genetic prediction of AD.

Levels of 1-ribosyl-imidazoleacetate (−6.62%), metabolonic lactone sulfate (2.90%), and nonadecanoate (19:0) (−12.17%) respectively mediate the total genetic predictive impact of class Actinobacteria on the risk of AD (Figure 7; Supplementary material S9).

Levels of 2-stearoyl-GPE (18:0) (−9.87%), octadecanedioylcarnitine (C18-DC) (4.44%), 1-(1-enyl-stearoyl)-2-oleoyl-GPE (p-18:0/18:1) (38.66%), and X-23639 (13.28%) respectively mediate the total genetic predictive impact of family Lactobacillaceae on the risk of AD (Figure 7; Supplementary material S9).

Hexadecanedioate (C16-DC) levels (5.45%) mediate the total genetic predictive impact of genus *Ruminiclostridium6* on the risk of AD (Figure 7; Supplementary material S9).

Indole-3-carboxylate levels (13.91%), X-13431 levels (7.08%), alpha-ketoglutarate to succinate ratio (−13.91%), 3-phosphoglycerate to glycerate ratio (15.27%), and succinate to proline ratio (−14.64%) respectively mediate the total genetic predictive impact of genus *Ruminiclostridium9* on the risk of AD (Figure 7; Supplementary material S9).

## 4 Discussion

In this genetic causal study, we have identified several common microbial taxa associated with an increased risk of AD. Specifically, we observed positive correlations between class Actinobacteria, family Lactobacillaceae, genus *Lachnoclostridium*, genus *Ruminiclostridium6*, and AD risk, while genus *Ruminiclostridium9* showed a negative correlation with AD risk. Mediation analysis based on MR suggests that certain metabolites may serve as potential mediators of the causal relationship between these microbial taxa and AD. For instance, lactone sulfate may act as a potential mediator of the causal relationship between class Actinobacteria and AD, while Octadecanedioylcarnitine (C18-DC) and 1-(1-enyl-stearoyl)-2-oleoyl-GPE (p-18:0/18:1) may serve as potential mediators of the causal relationship between family Lactobacillaceae and AD. Additionally, Hexadecanedioate (C16-DC) may act as a potential mediator of the causal relationship between genus *Ruminiclostridium6* and AD, and Indole-3-carboxylate and 3-phosphoglycerate to glycerate may serve as potential mediators of the causal relationship between genus *Ruminiclostridium9* and AD.

The human gastrointestinal tract harbors the largest and most complex microbial ecosystem, comprising microorganisms and the host microenvironment, including tissues, cells, and metabolites. This system plays crucial roles in various physiological and pathological processes such as metabolism, immune regulation, and endocrine modulation (Qu et al., 2021). Mounting evidence suggests the involvement of the gut microbiota in the onset and progression of AD (Kesika et al., 2021). Bacteria such as *Helicobacter pylori*, *Borrelia burgdorferi*, and *Chlamydia pneumoniae* have been implicated in AD susceptibility by promoting excessive phosphorylation of tau protein and elevating levels of pro-inflammatory bacteria (e.g., *Escherichia/Shigella*) while reducing levels of anti-inflammatory gut microbes (e.g., *Ruminococcus*) (Cryan et al., 2019; Murray et al., 2022). Moreover, several experimental studies conducted in AD mice have reported increased abundance of *Escherichia coli-Shigella* and *Desulfovibrio*, accompanied by elevated levels of *Enterobacteriaceae*, *Pseudomonas*, and *Clostridium*, which promote amyloid-like protein deposition in the brain, microglial cell accumulation, inflammatory responses, and contribute to the pathogenesis of AD.

Our MR study indicates that the increase in Actinobacteria, Lactobacillaceae, Lachnoclostridium, and *Ruminiclostridium6* is positively associated with the risk of AD, while a decrease in *Ruminiclostridium9* is negatively associated with the risk of AD. These microbiota may influence the gut-brain axis by modulating gut permeability, the immune system, or metabolism, thereby contributing to the development and progression of AD (Zhuang et al., 2018). However, previous research (Zhuang et al., 2018) has found a significant decrease in the abundance of Actinobacteria in AD patients. Additionally, Murray et al. (2022) reported that Lactobacillaceae might play a role in neuroinflammation and cognitive function, although the precise mechanisms remain unclear. Another study Zou et al. (2024) also suggested an association between Lactobacillaceae and cognitive impairment in AD patients. While our MR study found a negative correlation between *Ruminiclostridium9* and AD, and a positive correlation between *Ruminiclostridium6* and AD, Kesika et al. (2021) indicated that *Ruminiclostridium* plays an important role in the gut microbiota, but its specific functions and effects may vary across different studies. These discrepancies might first arise from differences in study design. Our study employed MR methods, emphasizing the use of genetic instrumental variables, whereas Zhuang et al. (2018) study relied on cross-sectional data. Cross-sectional studies depend on observational data, comparing data from different groups or time points to identify associations. Such designs are susceptible to confounding factors, as they cannot completely rule out all potential confounders, making these data more prone to influence by environmental and lifestyle factors, such as diet and living conditions. These confounders are challenging to control in cross-sectional studies, but MR analyses can largely mitigate these issues. Furthermore, the subjects in different studies might vary in race, age, sex, and health status, potentially influencing the relationship between gut microbiota and AD. Our MR study primarily used data from European populations, possibly leading to ethnicity-specific results. For example, Zhuang et al. (2018) study focused on Chinese populations, which might exhibit different gut microbiota compositions and associations with AD. Variations in genetic background and lifestyle across races can affect the diversity and function of gut microbiota, resulting in different study outcomes. Additionally, the incidence of AD and the composition of gut microbiota might vary by age and sex. For instance, elderly populations might have distinct gut microbiota structures, and sex differences could influence the relationship between gut microbiota and AD (Zou et al., 2024). Lastly, differences in microbial sequencing technologies, data processing, and analysis methods across studies could contribute to varying results. While 16S rRNA sequencing and whole-genome shotgun sequencing are commonly used to sequence gut microbiota, 16S rRNA sequencing primarily identifies and classifies bacteria but offers lower resolution, making it difficult to accurately identify species and strains (Durazzi et al., 2021). In contrast, whole-genome shotgun sequencing provides higher resolution data, identifying more microbial species and functional genes, but is costlier and more complex to analyze. Thus, studies may employ different data processing and analysis methods, such as OTU (operational taxonomic unit) clustering and ASV (amplicon sequence variant) analysis, which can impact the classification and abundance estimates of gut microbiota, leading to disparate results (Rausch et al., 2019).

It is now widely recognized that the gut microbiota plays a significant role in the development of AD. In addition to alterations in the composition and abundance of gut microbiota affecting AD, the interaction between the gut microbiota and the “brain-gut” axis can also impact the onset and progression of AD through the influence of biologically active metabolites (Zou et al., 2024). Genera such as Streptomyces, Bacillus, and Clostridium, along with their metabolites such as short-chain fatty acids, tryptophan, and glutamate, undergo changes in AD, manifested by Aβ accumulation, neuronal damage, and synaptic dysfunction (Cryan et al., 2020; Gubert et al., 2020). For instance, quinolinic acid, a biologically active metabolite derived from tryptophan degradation, is upregulated in the brain tissues of AD patients due to its lipid peroxidation and neurotoxicity (Frausto et al., 2021). Short-chain fatty acids, on the other hand, improve hippocampal neuroprotection and plasticity and reduce Aβ plaques in AD models by activating G protein-coupled receptors (GPCRs) to stimulate enteroendocrine L cells to release glucagon-like peptide-1 (GLP-1) (Dalile et al., 2019). Recent studies have demonstrated that vitamin D, gut microbiota, and their metabolites, short-chain fatty acids, synergistically regulate the immune system (Murdaca et al., 2021, 2024; Murdaca and Gangemi, 2023). Specifically, butyrate not only enhances the expression of the vitamin D receptor (VDR) but also potentiates the differentiation-inducing effects of 1,25-dihydroxyvitamin D3. The VDR plays a crucial role in the butyrate-mediated inhibition of NF-κB activation in human colon cancer cells (Malaguarnera, 2020). Furthermore, short-chain fatty acids and vitamin D exhibit a cooperative effect in enhancing the synthesis of host defense peptides (HDPs), which are integral components of the innate immune system with antimicrobial and immunomodulatory activities (Robinson et al., 2018). Our MR mediation analysis suggests that lactone sulfate may be a potential driver of the causal relationship between class Actinobacteria and AD. Octadecanedioylcarnitine (C18-DC) and 1-(1-enyl-stearoyl)-2-oleoyl-GPE (p-18:0/18:1) may similarly serve as potential drivers of the causal relationship between family Lactobacillaceae and AD. Hexadecanedioate (C16-DC) may act as a potential driver of the causal relationship between genus *Ruminiclostridium6* and AD. Additionally, indole-3-carboxylate and the 3-phosphoglycerate to glycerate ratio may represent potential drivers of the causal relationship between genus *Ruminiclostridium9* and AD.

Lactone sulfate is a steroid-like metabolite positively correlated with BMI, liver fat percentage, and visceral fat volume (Zheng R. et al., 2023). Although there is currently no direct literature linking lactone sulfate to AD, accumulating evidence suggests that dysregulation of endogenous steroid concentrations and their biosynthetic enzymes play significant roles in the pathogenesis of AD (Luchetti et al., 2011; Pike, 2017). Studies have found that pregnenolone sulfate (PREGS) and dehydroepiandrosterone sulfate (DHEAS) are significantly lower in elderly AD patients compared to age-matched non-demented controls, particularly in the striatum, cerebellum, and hypothalamus, and negatively correlated with high levels of cortical Aβ and phosphorylated tau proteins (Weill-Engerer et al., 2002), where PREGS and DHEAS are types of steroids. Considering that PREGS and DHEAS belong to the steroid class, it can be inferred that lactone sulfate may be negatively correlated with AD. This is consistent with our MR study results, where class Actinobacteria showed a positive correlation with AD, and the gene-predicted lactone sulfate mediated proportion reached 2.90%. Therefore, it can be speculated that in the process of class Actinobacteria affecting the onset and progression of AD, lactone sulfate may serve as a protective factor and a target for preventing AD, helping to mitigate the excessive impact of class Actinobacteria on the development of AD.

Octadecenoylcarnitine is a form of acylcarnitine categorized as long-chain acylcarnitines due to its acyl group containing 14 to 20 carbons, which is formed by the esterification of long-chain fatty acids ingested through diet (Dambrova et al., 2022). It plays a crucial role in transporting fatty acids into mitochondria in the human body (Reuter and Evans, 2012) and its levels variations serve as important indicators of inherited disorders of long-chain fatty acid metabolism, such as schizophrenia (Cao et al., 2020) and ischemia–reperfusion injury (Liepinsh et al., 2016). Recent research (Horgusluoglu et al., 2022) has revealed novel metabolites and potential regulatory factors in AD through integrated multi-omics data analysis, demonstrating the association between short-chain acylcarnitines/amino acids and medium−/long-chain acylcarnitines with AD clinical outcomes, and identifying the involvement of ABCA1 and CPT1A in the regulation of acylcarnitines and amino acids in AD. Specifically, L-carnitine, acetyl-L-carnitine, and propionyl-L-carnitine may act on mitochondrial function and mobility changes in neurons induced by amyloid-β peptide 1–42 oligomers (AβOs) in different ways, thereby alleviating AD-related pathology (Mota et al., 2021). According to the results of our MR study, family Lactobacillaceae showed a positive correlation with AD, with the gene-predicted octadecenoylcarnitine mediated proportion reaching 4.44%. Therefore, it can be speculated that in the process of family Lactobacillaceae affecting the onset and progression of AD, octadecenoylcarnitine may serve as a protective factor and a target for preventing AD.

In this MR study, it was found that certain intermediate metabolites, such as 1-(1-enyl-stearoyl)-2-oleoyl-GPE (p-18:0/18:1), Hexadecanedioate (C16-DC), Indole-3-carboxylate, and the 3-phosphoglycerate to glycerate ratio, have been relatively underexplored in their association with gut microbiota and AD. Therefore, these metabolites could be considered novel intervention targets. By delving into their mechanisms of action and interaction networks, further exploration of their potential roles in the interplay between gut microbiota and AD onset and progression is warranted. This offers new avenues and potential for future research endeavors. Recent studies have highlighted the potential of extracellular vesicles (EVs) as non-invasive biomarkers for various diseases (Li et al., 2018, 2019, 2020, 2021; Lai et al., 2022). EVs, including exosomes and microvesicles, are nanosized endocytic vesicles secreted by most cell types, carrying a rich cargo of proteins, lipids, and various RNA species (Li et al., 2018). Among these, long RNA species such as messenger RNA (mRNA), circular RNA (circRNA), and long non-coding RNA (lncRNA) are of particular interest due to their stability and abundance in blood (Li et al., 2019). Research has shown that these long RNAs in EVs can reflect the physiological and pathological state of their cells of origin, making them promising candidates for disease biomarkers (Li et al., 2019). The exoRBase database, for instance, has compiled extensive RNA-seq data of exosomal RNAs from human blood, providing valuable resources for identifying molecular signatures in various diseases, including cancer (Li et al., 2018; Lai et al., 2022). Furthermore, extracellular vesicle long RNA (exLR) profiles have been used to distinguish cancer patients from healthy individuals with high diagnostic accuracy, suggesting their potential utility in non-invasive disease diagnostics (Li et al., 2019, 2021). Given the complex interplay between the gut microbiota and AD, and the potential role of blood metabolites as mediators, it is plausible that exLR could serve as valuable biomarkers for AD. By reflecting the alterations in gut microbiota and associated metabolic changes, exLR might offer a novel approach for early diagnosis and monitoring of AD progression.

Our MR study possesses both strengths and limitations. Firstly, we conducted, for the first time, a mediation analysis of human serum metabolites predicted by genes in relation to gut microbiota and AD, thus fundamentally overcoming limitations inherent in traditional observational studies, such as environmental confounding and reverse causality due to inadequate sample sizes. Secondly, we utilized GWAS data with an ample number of cases and excluded weak instrumental variables, thereby enhancing statistical power. Additionally, we performed sensitivity analyses and employed various statistical models for repeated analyses to elucidate different multivariate patterns, thereby strengthening the evidence of our study findings. However, this MR study has several limitations. Firstly, the MR analysis is primarily based on European populations, and the findings may not be entirely applicable to other ethnic or regional groups. Differences in genetic background, environmental exposures, and lifestyle across various populations could affect the relationship between gut microbiota, metabolites, and AD, thus limiting the generalizability of the results. Secondly, potential confounding factors, such as comorbidities and medication use, were not fully accounted for. These factors might influence the relationship between metabolites and AD; for example, comorbidities could affect metabolite levels through shared metabolic pathways, and multiple comorbidities could lead to chronic low-grade inflammation, which may alter the gut microbiota composition and subsequently the metabolite profile, thereby contributing to AD development. Additionally, medications can indirectly influence gut microbiota and metabolite levels by altering inflammation status and oxidative stress. Moreover, we utilized AD data solely from PGC, which provides evidence for the association between gut microbiota, metabolites, and AD but lacks direct biological mechanism validation. Relying on a single data source or database may introduce bias, and cross-validation with multiple data sources can enhance the credibility of the results. Future research should aim to overcome these limitations and explore the potential roles of metabolites in the influence of gut microbiota on AD development more comprehensively and deeply, thereby uncovering the mechanisms underlying the gut microbiota-metabolite-AD axis and providing novel insights and approaches for AD prevention and treatment. First, large-scale, long-term cohort studies should be conducted to improve the statistical power and reliability of the findings. These studies should include diverse regions and ethnicities to validate the generalizability of the results and to investigate the impact of regional and ethnic differences. Second, collaborative multi-center studies should be undertaken to aggregate data from different sources, thereby increasing the sample size and data diversity, which would enhance the representativeness and generalizability of the findings. Third, confounding factors such as comorbidities and medication use should be included, with multivariable adjustments made to minimize their interference. Sensitivity analyses should be performed to assess the stability of the results under different comorbidity and medication use scenarios. Finally, both *in vitro* and *in vivo* experiments should be conducted to elucidate the biological mechanisms through which gut microbiota and metabolites influence AD.

## 5 Conclusion

In conclusion, our bidirectional two-sample mediation MR analysis provides genetic evidence indicating a positive correlation between class Actinobacteria, family Lactobacillaceae, genus *Lachnoclostridium*, genus *Ruminiclostridium 6*, and the risk of AD, while genus *Ruminiclostridium 9* exhibits a negative correlation with AD risk. Lactone sulfate may serve as a potential driving factor for the causal relationship between class Actinobacteria and AD, while Octadecanedioylcarnitine (C18-DC) and 1-(1-enyl-stearoyl)-2-oleoyl-GPE (p-18:0/18:1) could potentially drive the association between family Lactobacillaceae and AD. Additionally, Hexadecanedioate (C16-DC) might play a role in the relationship between genus *Ruminiclostridium 6* and AD, whereas Indole-3-carboxylate and 3-phosphoglycerate to glycerate may influence the association between genus *Ruminiclostridium 9* and AD. Subsequent steps should involve further research extending the study population to East Asian or other regions to better delineate the potential role of human serum metabolites in the interplay between gut microbiota and AD. Future endeavors should entail the utilization of serum metabolomics to sample and analyze AD patients in randomized controlled trials using specific serum metabolites identified in this MR analysis, including lactone sulfate, Octadecanedioylcarnitine (C18-DC), 1-(1-enyl-stearoyl)-2-oleoyl-GPE (p-18:0/18:1), Hexadecanedioate (C16-DC), Indole-3-carboxylate, and 3-phosphoglycerate to glycerate. Moreover, macrogenomic sampling of AD patients in randomized controlled trials should be conducted to validate the reliability and authenticity of our MR study results.

## Data availability statement

The gut microbiota data provided in the study is stored in MiBioGen (https://mibiogen.gcc.rug.nl/). The Alzheimer’s disease data provided in the study is stored in The Psychiatric Genomics Consortium (PGC) (https://pgc.unc.edu/for-researchers/download-results/). The human blood metabolite data provided in the study is saved in the GWAS Catalog (https://www.ebi.ac.uk/gwas/publications/36635386), accession numbers GCST90199621-GCST9021020.

## Ethics statement

All individual participants in the study provided informed consent.

## Author contributions

GC: Conceptualization, Data curation, Formal analysis, Writing – original draft, Writing – review & editing. YJ: Formal analysis, Investigation, Methodology, Writing – review & editing. CC: Methodology, Project administration, Software, Writing – review & editing. YZ: Methodology, Software, Writing – review & editing. YC: Software, Supervision, Validation, Writing – review & editing. XZ: Validation, Visualization, Writing – review & editing.
